# Arctic grayling (*Thymallus arcticus*) in saltwater: a response to Blair *et al*. (2016)

**DOI:** 10.1093/conphys/cow055

**Published:** 2016-11-16

**Authors:** Kurt C. Heim, Matthew S. Whitman, Lawrence L. Moulton

**Affiliations:** 1 Ecology Department, 310 Lewis Hall, PO Box 173460, Montana State University, Bozeman, MT 59717, USA; 2 US Bureau of Land Management, Arctic Office, 222 University Avenue, Fairbanks, AK 99709, USA; 3 Owl Ridge Natural Resource Consultants, 1012 Shoreland Drive, Lopez Islands, WA 98261, USA

## Abstract

Although not well known, Arctic grayling can move through saline waters and are captured regularly in nearshore coastal waters in Arctic Canada and Alaska with salinities up to 18 ppt. We highlight the implications this has for Blair *et al*. (2016), a paper recently published in *Conservation Physiology*.

## Introduction

Blair *et al*. (2016) present a laboratory trial that clearly demonstrates the reduced salinity tolerance of Arctic grayling (*Thymallus arcticus*) compared with rainbow trout (*Oncorhynchus mykiss*). While rainbow trout exposed to 17 ppt salinity were able to adjust osmoregulation successfully and establish homeostasis, Arctic grayling showed increasing serum sodium and chloride over time, which eventually led to mortality. They conclude that the probable impacts of highly saline water spills or discharge into freshwater habitats should be based on species-specific salinity tolerances, rather than the response of the euryhaline (able to tolerate a range of salinities) rainbow trout (Environment Canada, 1990; USEPA, 2002). This is especially important as oil and gas development continues across Alaska and Canada.

We agree with the main conclusions and conservation implications of this article but are writing here to address an inaccuracy. Arctic grayling are described as having a ‘strict(ly) freshwater existence’ (pp 1, 5 and 9), and the authors suggest that ‘Arctic grayling have lost the ability to execute the necessary osmoregulatory mechanisms to cope with higher salinity environments.’ Although Arctic grayling is indeed a freshwater species, there is evidence that some populations can tolerate saline waters. A ‘strictly freshwater existence’ would preclude the use of any brackish (0.5–29 ppt) habitats; however, in the Arctic this species is known to move through brackish waters during migration (West *et al*., 1992) in coastal watersheds, and is routinely captured in samples collected in brackish estuaries (Moulton and Fawcett, 1984; Craig *et al*., 1985; Wiswar and West, 1987; Fruge *et al*., 1989; Bond and Erickson, 1992; Griffiths *et al*., 1998; Roux *et al*., 2016). It is not widely recognized that Arctic grayling use saline waters, and it is our intent to provide a brief introduction to this concept and explore the implications for Blair *et al*. (2016).

## Arctic grayling in saltwater

The most well-known example of Arctic grayling using saline waters comes from a radio telemetry study conducted on the Arctic Coastal Plain of Alaska (West *et al*., 1992). In this harsh aquatic environment, where most bodies of water <1.5 m deep freeze solid (Jones *et al*., 2009), annual migrations by Arctic grayling can involve travel through estuarine waters. Adult Arctic grayling were found migrating in the autumn from several drainages, through brackish waters and into the HulaHula River to overwinter. This study clearly demonstrates successful travel through saline waters by Arctic grayling.

Arctic grayling are routinely—albeit in low densities—captured in nearshore coastal waters off the northern coast of Alaska (Moulton and Fawcett, 1984; Craig *et al*., 1985; Wiswar and West, 1987; Fruge *et al*., 1989; Griffiths *et al*., 1998) and Canada (Bond and Erickson, 1992; Roux *et al*., 2016). It is presumed that Arctic grayling will enter brackish lagoons with salinities <4 ppt to forage or move between river drainages (West *et al*., 1992; Griffiths *et al*., 1998). Within-summer forays into these habitats tend to occur earlier in the open-water season, when salinity levels are lower (Moulton and Fawcett, 1984; Griffiths *et al*., 1998), and across habitats within an estuary the abundance of Arctic grayling is negatively associated with salinity (Roux *et al*., 2016). Four ppt does not appear to be the upper salinity limit of Arctic grayling tolerance; Moulton and Fawcett (1984) report catches of Arctic grayling off Oliktok Point in the Arctic Ocean (70.509°, −149.863°) in waters up to ~18 ppt. How extensive these forays and migrations may be is unknown; however, they contradict the statements of Blair *et al*. (2016) citing the ‘strict freshwater existence [of Arctic grayling] in North American for the past 3–5 million years.’

The overall contribution of saline water use to coastal Arctic grayling populations and production is not known, and it is important to recognize that habitat use does not prove habitat importance (Rabeni and Sowa, 1996). It may be that the presence of Arctic grayling in estuarine waters is, in some cases, incidental, resulting from fish being swept downstream during snowmelt runoff in the spring. Estuaries could act as a population sink if fish could not return to waters of suitable salinity. However, during the early spring the estuarine waters are at an annual salinity low and could provide an important dispersal corridor between drainages. The near-shore marine environment may also provide abundant food resources; other salmonids that overwinter in Arctic rivers for ~9 months move specifically to the marine environment during summer to feed on invertebrates (Craig *et al*., 1985; Fechhelm *et al*., 1995). If these movements are indeed common, widespread or important, it raises new conservation concerns regarding the potential anthropogenic disturbances to this behaviour.

## Implications for Blair *et al*. (2016)

Local adaptation to salinity tolerance is common in salmonids and other genera of fishes (Schulte, 2007; Larsen *et al*., 2008); however, no such comparison has been made across coastal and inland populations of Arctic grayling. Therefore, it is unclear to what extent the results of Blair *et al*. (2016) are influenced by their use of specimens from the landlocked providence of Alberta. The differences in osmoregulation abilities across a species range can be great; for example, after transfer to freshwater, specimens from a northern population of common killifish (*Fundulus heteroclitus*) experienced very little mortality, whereas 19% of fish from a southern population died (Scott *et al*., 2004). Local adaptation can indeed alter the molecular response of gills to salinity variation (Scott and Schulte, 2005). Furthermore, the closely related European grayling (*Thymallus thymallus*) has evolved an anadromous form in several locations in the Baltic Sea (Northcote, 1993; Koskinen *et al*., 2000; Swatdipong *et al*., 2009), demonstrating the adaptive potential within *Thymallidae.* Finally, we would also like to point out that anadromous forms of lake trout (*Salvelinus namaycush*)—previously considered the least tolerant salmonid to saltwater—have recently been discovered (Swanson *et al*., 2010), and perhaps not coincidentally, this discovery was made in the Arctic.

Although the specimens used by Blair *et al*. (2016) were members of the species Arctic grayling, discretion should be used when extrapolating results of individuals to their species. Perpetuating a ‘strict freshwater existence’ of Arctic grayling could stifle future research and discovery regarding their physiology, behaviour and population dynamics. Although we do not expect Arctic grayling to have comparable salinity tolerance to rainbow trout, we suggest that coastal populations may show local adaptations that allow use of higher-salinity waters. Replicating the trials of Blair *et al*. (2016) with Arctic grayling sourced from coastal watersheds could shed light on this uncertainty.

In conclusion, we would like to acknowledge again the well-designed and -conducted study of Blair *et al*. (2016) with regard to their main objectives. We agree with the conservation implications of Blair *et al*. (2016); the anticipated effects of highly saline water spills into native salmonid habitats should not universally be judged with a euryhaline species, such as the rainbow trout. We hope that this response will lead to an increased understanding of Arctic grayling using saltwaters in coastal regions, as well as entice future research to describe the relative importance and prevalence of this potentially adaptive behaviour.

## Supplementary Material

Supplementary Data
