# Supplementary material for: Arctic grayling (Thymallus arcticus) in saltwater: a response to Blair et al. (2016)
Source: Conserv Physiol. 2016 Nov 16;4(1):cow055. doi: 10.1093/conphys/cow055 (PMC5142049; doi:10.1093/conphys/cow055)
Supplement: Supplementary Data [file supp_4_1_cow055__index.html]

Supplementary Data 

# Arctic grayling (*Thymallus arcticus*) in saltwater: a response to Blair *et al*. (2016)

## Supplementary Data

Supplementary Data

- Supplementary Data - docx file
