# Supplementary material for: Arctic grayling (Thymallus arcticus) in saltwater: a response to Blair et al. (2016)
Source: Conserv Physiol. 2016 Nov 16;4(1):cow055. doi: 10.1093/conphys/cow055 (PMC5142049; doi:10.1093/conphys/cow055)
Supplement: Supplementary Data [file supp_cow055_Goss_comment.docx]

Response from Goss, Blair et al to Heim comment

We would like to thank Heim and colleagues for their interest and reply to our study (Heim et al. 2016). Whilst we agree that, as a species, Arctic grayling (*Thymallus arcticus*) should perhaps be considered a freshwater stenohaline fish, the intention of our study was to contrast the behavior of the freshwater form of the species, which is threatened by spills of industrial saline waters, with the anadromous life history of the rainbow trout (*Oncorhynchus mykiss*), which is used as a keystone regulatory species for environmental impacts (Blair et al. 2016).

Further studies on the migration of the Arctic grayling through brackish waters (<4ppt) and their feeding patterns during these excursions are clearly necessary. Local adaptation to salinity is possible and genetic divergence theories, similar to those we invoke to understand the differences in salinity tolerance between rainbow trout and freshwater populations of Arctic grayling may apply to different populations of Arctic grayling. Overall, we agree that a more complete understanding of grayling biology and physiology is needed to properly protect and manage these relatively understudied salmonids in the face of Northern development.

Blair SD, Matheson D, He Y, Goss GG (2016) Reduced salinity tolerance in the Arctic grayling ( *Thymallus arcticus* ) is associated with rapid development of a gill interlamellar cell mass : implications of high-saline spills on native freshwater salmonids. *Conserv Physiol* 1–11

Heim KC, Whitman MS, Moulton LL (2016) Arctic grayling (*Thymallusarcticus*) in saltwater: a response to Blair et al. 2016. *Conserv Physiol*
